# Bacteriophage-Based Biosensors: A Platform for Detection of Foodborne Bacterial Pathogens from Food and Environment

**DOI:** 10.3390/bios12100905

**Published:** 2022-10-21

**Authors:** Rashad R. Al-Hindi, Addisu D. Teklemariam, Mona G. Alharbi, Ibrahim Alotibi, Sheren A. Azhari, Ishtiaq Qadri, Turki Alamri, Steve Harakeh, Bruce M. Applegate, Arun K. Bhunia

**Affiliations:** 1 Department of Biological Sciences, Faculty of Science, King Abdulaziz University, Jeddah 21589, Saudi Arabia; 2 Health Information Technology Department, Applied College, King Abdulaziz University, Jeddah 21589, Saudi Arabia; 3 Family and Community Medicine Department, Faculty of Medicine in Rabigh, King Abdulaziz University, Jeddah 21589, Saudi Arabia; 4 King Fahd Medical Research Center, Yousef Abdullatif Jameel Chair of Prophetic Medicine Application, Faculty of Medicine, King Abdulaziz University, Jeddah 21589, Saudi Arabia; 5 Department of Food Science, Purdue University, West Lafayette, IN 47907, USA; 6 Purdue Institute of Inflammation, Immunology and Infectious Disease, Purdue University, West Lafayette, IN 47907, USA; 7 Interdisciplinary Life Science Program (PULSe), Purdue University, West Lafayette, IN 47907, USA; 8 Department of Comparative Pathobiology, Purdue University, West Lafayette, IN 47907, USA

**Keywords:** bacteriophage, biosensor, detection, food, water, pathogenic bacteria

## Abstract

Foodborne microorganisms are an important cause of human illness worldwide. Two-thirds of human foodborne diseases are caused by bacterial pathogens throughout the globe, especially in developing nations. Despite enormous developments in conventional foodborne pathogen detection methods, progress is limited by the assay complexity and a prolonged time-to-result. The specificity and sensitivity of assays for live pathogen detection may also depend on the nature of the samples being analyzed and the immunological or molecular reagents used. Bacteriophage-based biosensors offer several benefits, including specificity to their host organism, the detection of only live pathogens, and resistance to extreme environmental factors such as organic solvents, high temperatures, and a wide pH range. Phage-based biosensors are receiving increasing attention owing to their high degree of accuracy, specificity, and reduced assay times. These characteristics, coupled with their abundant supply, make phages a novel bio-recognition molecule in assay development, including biosensors for the detection of foodborne bacterial pathogens to ensure food safety. This review provides comprehensive information about the different types of phage-based biosensor platforms, such as magnetoelastic sensors, quartz crystal microbalance, and electrochemical and surface plasmon resonance for the detection of several foodborne bacterial pathogens from various representative food matrices and environmental samples.

## 1. Introduction

Foodborne microorganisms are an important cause of human illnesses worldwide. Two-thirds of human foodborne diseases are caused by bacterial pathogens throughout the globe, especially in developing nations [1]. The most commonly encountered foodborne bacterial pathogens are *Staphylococcus aureus* (*S. aureus*), *Salmonella enterica* serovar Typhimurium (*S.* Typhimurium), *Clostridium perfringens* (*C. perfringens*), *Campylobacter* species, *Escherichia coli* (*E. coli*), and *Listeria monocytogenes* (*L. monocytogenes*). Most of these organisms have zoonotic importance, causing huge adverse effects to both public health and economic sectors [1]. Of these bacterial foodborne pathogens, human-sourced pathogens such as *E. coli* and *Salmonella* Typhi can contaminate the food supply chain through the feces of infected individuals [2], while many others such as non-typhoidal *Salmonella*, *Campylobacter*, *Staphylococcus*, *Yersinia*, *Clostridium*, and *Listeria* are transmitted through food animals, poultry, milk, or eggs [3]. Environmental transmission has been frequently reported for several of the pathogens, including *Salmonella*, *E. coli* O157:H7, and *Campylobacter*, during pre- and post-harvest food processing, storage, and transportation [4]. The Centers for Disease Control and Prevention (CDC) routinely monitors the presence of these pathogens in food [5]. The US FDA (Food and Drug Administration) and FSIS (Food Safety Inspection Service) agencies strictly regulate their presence in raw or ready-to-eat products [5]; therefore, reliable detection methods that are capable of detecting live pathogens are critical.

Conventional foodborne pathogen detection methods mainly depend on specific biochemical, serological, and nucleic-acid-based techniques [6,7]. These methods require skilled technicians and are time-consuming, expensive, and difficult to interpret. Most rapid detection methods cannot distinguish dead from live cells unless a growth-based enrichment step is used, making them inapplicable in many food processing facilities [8]. Conversely, enzyme-linked immunosorbent assays (ELISA) or lateral flow immunochromatographic assays are simple and rapid biochemical immunoassays, but they have a low sensitivity [9,10]. Similarly, polymerase chain reaction (PCR), biochips, and microarrays are some, but not all, of the nucleic-acid-based techniques that have been used for the investigation of foodborne microbes [6,9,11]. Nevertheless, various types of PCR techniques such as reverse transcriptase and multiplex PCR are ineffective at processing a large volume of samples without a pre-enrichment step and have high processing costs that make them impractical for day-to-day use [12].

**Figure 1:**
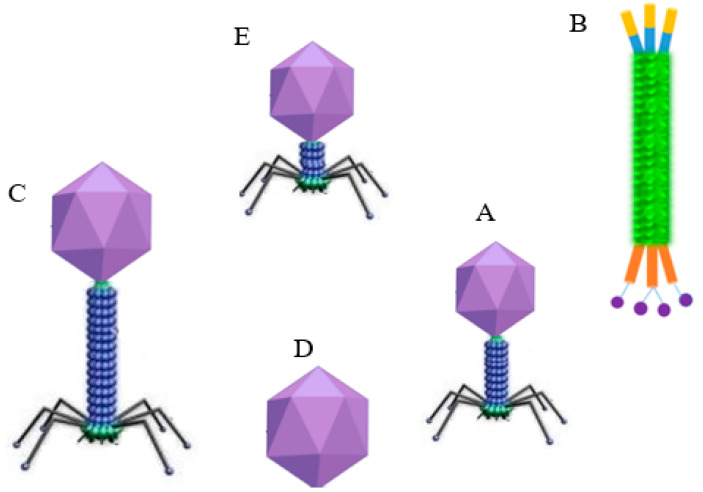
Schematic diagram showing the structure of major phage families: (**A**) *Myoviridae* (e.g., T4); (**B**) filamentous *Inoviridae* (e.g., M13); (**C**) long and noncontractile *Siphoviridae* (e.g., λ phage); (**D**) *Leviviridae*; and (**E**) short *Podoviridae* (e.g., T7).

Over the last few decades, bacteriophage-based biosensors have been recognized as a promising platform for detecting pathogens or sensing various biological analytes. Compared to other bio-receptors such as aptamers and antibodies, bacteriophages provide quite a few advantages in the detection of pathogens. Firstly, phages have a unique structure, including tail fibers that aid their binding to bacterial hosts, are highly specific, and are harmless to human cells (Figure 1). Virulent phages take 1–2 h to complete the infection cycle, quickening the release of the cytoplasmic marker from the infected host to be used in numerous detection systems. In addition, phages are the most abundant biological entities and are found in places where their host organism exists. They are relatively stable under various conditions, such as pH, temperatures, and organic/inorganic solvents, and they resist proteases. They are also cheaper to produce than antibodies and have a relatively long shelf life. It is easier to distinguish dead from live bacterial cells using this platform, as phages replicate only inside living bacteria [12].

The short shelf life of food products and the low infectious dose of most foodborne pathogens [13] are the most critical driving forces that push researchers to design sensitive, specific, and reliable detection techniques. The development of phage-based biosensors as a tool for the direct detection of live pathogens in food is an important and attractive approach [14]. Presently, several phage-based biosensors have been developed that incorporate various transducers, including electrochemical [15], quartz crystal microbalance (QCM) [16], surface plasmon resonance (SPR) [17], magnetoelastic (ME) [18], and others. Most of these biosensors have been designed using the whole/intact phage or the phage proteins as well as the cytoplasmic markers that are released following the phage infection. The performance of these biosensors varies, as they employ different immobilization methodologies (physical, chemical, covalent, or oriented) and/or transducers.

Efforts have been made in the last decade to optimize biosensor systems, including phage-based sensors, to enhance the reliability of the technique. As far as we know, phage-based biosensors for monitoring water and food samples have not yet been commercialized; however, the current trends show promise. This review provides an overview of the different types of phage-based biosensors and their application in the detection of foodborne bacterial pathogens, with a special emphasis on recently developed biosensor platforms.

## 2. Phage-Based Biosensors

According to the International Union of Pure and Applied Chemistry (IUPAC), the biosensor is defined as a self-controlled derivative material that contains a bio-recognition component (bio-receptor/bio-probe) linked to a transducer (sensor) to convert the biological signal into a digital signal in the computer system for interpretation [19]. Phage-based biosensor platforms generally consist of the network of the whole phage or partial phage particle, infection of the host bacterium, and finally production of colorimetric, electrical, fluorescent, or luminescent signals [20,21,22].

Lytic bacteriophages are primarily classified under the order *Caudovirales* (Figure 1) and are the principal biorecognition entities used as probes for phage-based biosensors. Apart from lytic phages, temperate phages also play a comparable role in the development of phage-based biosensors. Both lytic and temperate phages, such as HK620, P22, and ΦV10, have been used to develop reporter (engineered) phages [14]. Reporter phages are genetically modified by incorporating a reporter gene sequence into the phage genome to generate a measurable signal inside the intact host cell without killing (lysing) the host cell for the detection of live pathogens [14]. Moreover, proteins such as phage receptor-binding proteins (RBPs) have been recognized to be efficient bio-probes for replacing antibodies or other biomolecules, and have been used in the design of various types of biosensors [23]. In comparison to the whole phages, RBPs provide better stability across a broad range of pH values, temperatures, and gastrointestinal proteases [24]. Remarkably, appropriate tags (amino acids, e.g., cysteine) can be added to the RBP sequence at a specific site without affecting the binding ability and can be employed for the oriented surface functionalization of the RBPs on the biosensor platforms [24].

Bacteriophage-based biosensors offer several benefits for rapid bacterial detection [25]. They are highly specific towards their host organism, resist high temperatures (90–97 °C), and are stable across a wide range of pH values (3–14) and organic solvents. In comparison to antibodies, phages can be produced in large quantities easily and cheaply. They are eco-friendly and safe to use since they do not infect humans [26]. These characteristics make phages a novel bio-recognition tool for the development of biosensors for the detection of foodborne bacterial pathogens [27,28].

Today, phage-mediated biosensors have been developed as novel diagnostic tools in which specific phages are fixed to the device’s surface and then enabled to detect the analyte found in the sample [29]. Bacteriophages can be immobilized on a solid material with the aid of chemical, physical, or other immobilization or tethering techniques. The capture of targeted bacterial cells by surface-immobilized virions is an event that ends up with specific detection. The detection of pathogens using phage-based sensors is not limited to clinical samples, but is also used in a wide range of nonclinical applications, including foodborne pathogens from water and various food matrices [30], such as milk [31] and other perishable and non-perishable foodstuffs [32].

## 3. Phage-Mediated Bacterial Detection Approaches

### 3.1. Bacterial β-D-Galactosidase

**Figure 2:**
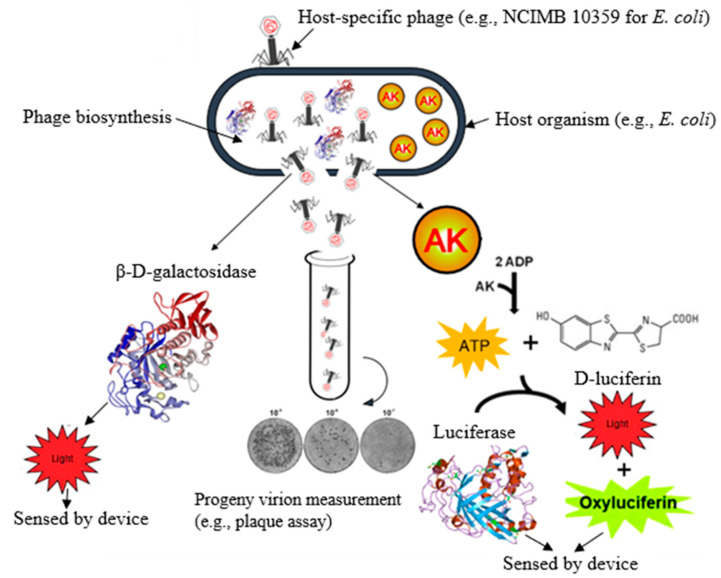
Schematic diagram showing the various intracellular cytoplasmic factors (biomarkers) released following phage infection. Abbr.: AK, adenosine kinase; ATP, adenosine triphosphate.

Lytic phages have been used for the detection of bacteria relying on the cytoplasmic contents (cell markers) released from the lysed cells (Figure 2). Neufeld and co-workers developed an amperometric assay based on bacterial β-D-galactosidase activity to detect *E. coli* at a concentration of 1 CFU/100 mL within 6 to 8 h [33]. In this assay, β-D-galactosidase was released from the phage-infected host cell following lysis, and an externally added substrate, p-aminophenyl-β-D-galactopyranoside, was converted into p-aminophenol, whose successive oxidation could be sensed by a potentiostat-based device. Sample filtration and pre-incubation before phage infection have improved the sensitivity of the test. Yemini and co-workers reported two cytoplasmic markers for the detection of *Bacillus cereus* (*B. cereus*) and *Mycobacterium smegmatis* (*M. smegmatis*) with a detection limit of 10 CFU/mL using α- and β- glucosidase, respectively, within 8 h [34]. Similarly, the presence of *E. coli* in water has been detected after phage lysis with a detection limit of 40 CFU/mL in 8 h [35].

### 3.2. Adenosine Triphosphate

Adenosine triphosphate (ATP) is one of the cytoplasmic markers most extensively used for estimating the number of bacterial cells in a sample (Figure 2). The concentration of ATP in a live, average-sized bacterium is nearly 10^−15^ g and near-constant for different species, so the quantification of the concentration of ATP released via a bioluminescent assay enables us to determine the viable cell counts [36]. ATP drives the catalytic reaction of the luciferase enzyme, which converts luciferin into oxyluciferin aerobically, together with adenosine monophosphate (AMP), carbon dioxide, and pyrophosphate, ultimately emitting light at a level corresponding to the specific concentration of ATP [37]. The high amount of ATP found in many foodstuffs is one of the main drawbacks of this assay, which results in high detection limits ranging from 10^4^ to 10^5^ CFU/mL [38]. However, this problem could be addressed using a phage-based biosorbent (e.g., T4 phage) by concentrating the host organism on the filter surface, which has shown a significant improvement in assay sensitivity with a detection limit as low as 6 × 10^3^ CFU/mL within 2 h (Disruptor^TM^ filter) [39]. This assay is robust and highly accurate with a 60-fold higher concentration of the sample background flora than the concentration of host pathogens [39].

### 3.3. Adenylate Kinase

Adenylate kinase (AK) is a bacterial cytoplasmic marker released from phage-infected cells, and the assay developed based on this marker could be used as an alternative approach to enhance the sensitivity of the bioluminescent ATP assay [28,40]. Adenylate kinase is an enzyme that enhances ATP production in the presence of a high amount of adenosine diphosphate (ADP) [41]. Under optimal conditions, its sensitivity can be enhanced by the addition of ADP, where the detection limits of *Salmonella* and *E. coli* were lower than 10^3^ CFU/mL [42]. This technique has been improved by incorporating an immunomagnetic separation (IMS) system in which antibody-coated magnetic beads are used to capture the target organism, which is then purified and concentrated [43]. Variations of this approach have been developed for the detection of *Salmonella*, *Listeria*, *E. coli* O157, and other bacterial pathogens.

### 3.4. Conductivity (Impedance)

The conductivity of the microbial growth medium can be changed by the perpetuation of microbes in the medium via the transformation of small to large charged and uncharged metabolites. Bacteriophages are appropriate tools for the detection of bacterial impedance (the resistance to the current flow via the conducting medium) since the presence of phage in a sample causes the retardation of impedance in the presence of the host organism. Chang and colleagues have detected *E. coli* O157:H7 without changing the conductivity of the MacConkey-sorbitol medium in the presence of an anti-*E. coli* O157:H7 phage (AR1) [44]. The obvious challenge of direct conductivity-based detection techniques is the necessity of an appropriate culture medium optimized for measuring the impedance, in which development is usually labor-intensive and vulnerable to bacterial contamination with the background flora. Besides, not all target bacterium release charged metabolites, which may adversely affect the impedance and conductivity measurements. Some of these problems can be overcome by employing indirect impedimetric techniques, in which metabolites such as carbon dioxide released into the medium during the cultivation of the target bacterium can be removed by the addition of potassium hydroxide to facilitate impedance measurements [45]. This method is highly specific and sensitive, and has been utilized for the detection of many foodborne pathogens, such as *L. monocytogenes*, *S. aureus*, *Salmonella enterica*, *Campylobacter* species, *E. coli*, and *Enterococcus faecalis* [46].

### 3.5. Whole-Phage or Progeny Virion Detection

Lytic phages infect the host cell, and the number of progeny virions released from the infected cell is directly proportional to the number of bacteria infected. This approach was first reported by Stewart et al. (1998) [47], in which cells were infected with phages followed by treatment with a virucidal agent to eliminate the added phage, thus allowing only progeny phage to be detected. The developed assay was sensitive and could obtain results in 4 h using plaque assays. Alternative assays, including molecular diagnostic tools such as quantitative PCR (qPCR), have been used to determine the number of progeny virions released from the infected cells [38] as well. For instance, *B. anthracis* was detected by immuno-chromatography, which has been designed based on a lateral-flow assay and the amplification of the gamma phage (γ) in bacterial cells. The virions released from the infected cells have been detected via reporters made of polystyrene nanoparticles linked to anti-γ phage antibodies. The detection limit has been recorded as 2.5 × 10^4^ CFU/mL with a 2–4 h assay time [48].

The plaque assay is one of the easiest/most straightforward methods for detecting foodborne pathogens to determine infection by increased titer [49]. If the titer of the phages rises, it relates to the effective binding or adsorption of phages to the host bacteria, resulting in lysis and the release of progeny virions, and thus indicating the existence of the viable target pathogens in the food matrices as initially described by Stewart et al. [47]. Recently, an assay was developed that employed phages coupled with qPCR for the detection of *S. enterica* ser Enteritidis in spiked chicken meat samples [50]. Approximately 0.22 fg/µL of pure phage (vB_SenS_PVP-SE2) DNA and nearly10^3^ pfu/mL of virions were detected using the combined technique with a detection limit of <10 CFU/25 g for 10 h of analysis, which included 3 h pre-enrichment, 6 h co-incubation, and 1 h DNA enrichment and qPCR.

Despite its benefits, intact phages suffer from certain limitations that restrict their use in the development of whole-phage sensor systems. The fast adsorption of phages onto the host cell and their subsequent lytic activity may destroy the target bacterium before the completion of downstream detection steps. The size of phages is also another constraint that adversely affects the whole-phage detection system. Besides, some phages produce catalytic enzymes towards the receptors situated on the surface of the bacterial cell. For instance, the endorhaminosidase enzymes produced by the P22 phage can degrade the O-antigen of the outer membrane structure of Gram-negative bacteria, especially *Salmonella enterica*, which then affects the subsequent attachment process. The *S. flexneri* phage, Sf6, shows similar endorhamnosidase-mediated cleavage [51]. Such phage-encoded enzymes can interfere with biosensor performance, leading to poor signal output. Moreover, intact phages can dry up on the surface of the biosensor, which ultimately can collapse and prevent tail fibers from attaching to the target bacterium [52].

### 3.6. Reporter Phages

Reporter bacteriophages are also engineered to integrate/insert a specific gene into the host bacterial genome to facilitate the visualization and subsequent detection of the host bacterium. Both lytic and lysogenic bacteriophages have been used for this purpose [14]. Currently, three types of phage engineering approaches have been reported: direct cloning, homologous recombination, and whole-genome activation. Reporter phages are designed to enable the detection of pathogens based on the enzymatic conversion of a chromogenic substrate [53]. Several reporter phages have been developed for the detection of foodborne bacteria. For instance, T7-ALP [54], Φ V10 lux [55], ΦV10 NanoLuc luciferase (Nluc) [56], T7-NRGp5 [57], and T4-NRGp17 [58] have been developed for the detection of different *E. coli* strains from various food matrices [59].

### 3.7. Phage-Associated Proteins

Phage receptor-binding proteins (RBPs) are the most variable structures of phages, which are responsible for recognizing specific receptors on the host bacterium [60]. Unlike antibodies, these proteins are relatively resistant to a wide range of pH values and heat treatments as well as protease activity, while showing analogous or even superior specificity [61]. These intrinsic features make RBPs more efficient and much-needed biorecognition elements for the specific and rapid detection of bacterial pathogens from different matrices [61]. These specialized phage binding proteins have been used for the detection of pathogens such as *Shigella* [62], *Salmonella* [63], and *P. aeruginosa* [64] from different food samples. Similarly, Poshtiban and co-workers designed magnetic beads by immobilizing the RBP protein Gp047, derived from the phage NCTC12673, and used them for the capture and detection of *Campylobacter* from chicken broth and milk samples [65].

Cell wall-binding domains (CBDs) of bacteriophage-encoded peptidoglycan hydrolases, commonly called endolysins, are the other phage-associated proteins (polypeptides) that have a high affinity and specificity towards the ligands on the Gram-positive cell wall [66]. Currently, CBD-based magnetic separation (CBD-MS) has been effectively used for detecting several Gram-positive foodborne bacteria, such as *B. cereus* [67], *Listeria* [66], and *Clostridium tyrobutyricum* [68].

## 4. Phage Immobilization Strategies

**Figure 3:**
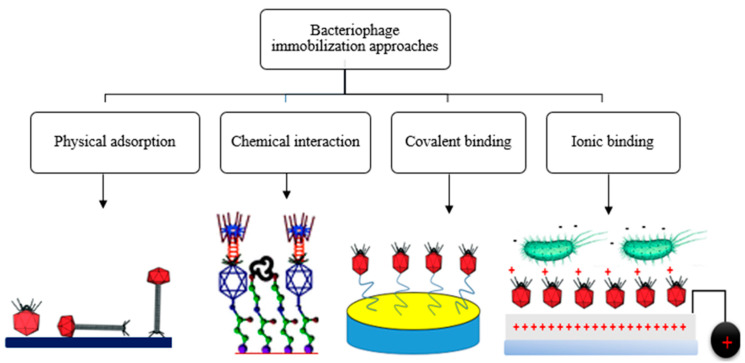
Strategies used for immobilization of phages on biosensor platforms.

Bacteriophage immobilization is the principal factor that determines the efficient detection of bacterial pathogens on a specific platform [69]. Various strategies have been established for the immobilization of phages on the electrode surface (Figure 3). The major phage immobilization techniques on solid surfaces include physical adsorption [70], covalent bonding [71], chemical interaction, and many more [72].

Physical adsorption is one of the easiest immobilization approaches for phages on a solid surface [70]. This approach involves the minimal use of chemicals, wherein phages are arranged randomly unless a surface and/or phage modification is performed. In this technique, the adsorbed phage may detach from the surface of the substrate due to changes in temperature, pH, or ionic concentrations, thus affecting biosensing performance [70]. Chemical-mediated immobilization approaches may cause the partial inactivation of the phage, most likely due to the alteration of domains involved in the interaction between the bacteriophage and the host cell’s surface. This approach, however, cannot guarantee the proper orientation of immobilized phages unless the immobilization approach is modified. The covalent interaction of phages on the surface of the substrate provides a firm binding and low risk of detachment of phages from the substrate. This technique produces a sufficient phage mass, which is required for phage application in the development of biosensors [73].

## 5. Types of Phage-Based Biosensors

### 5.1. Phage-Based Optical Biosensors

Optical biosensors are one of the best diagnostic tools for detecting pathogenic bacteria because of their high compatibility and sensitivity. Optical biosensors are developed by taking advantage of different properties of light such as wavelength, polarization, and the refractive index [74]. The most commonly employed optical phage-based detection techniques are chemo/bioluminescence, fluorescence spectrometry, and SPR (Table 1).

#### 5.1.1. Surface Plasmon Resonance Sensors

**Figure 4:**
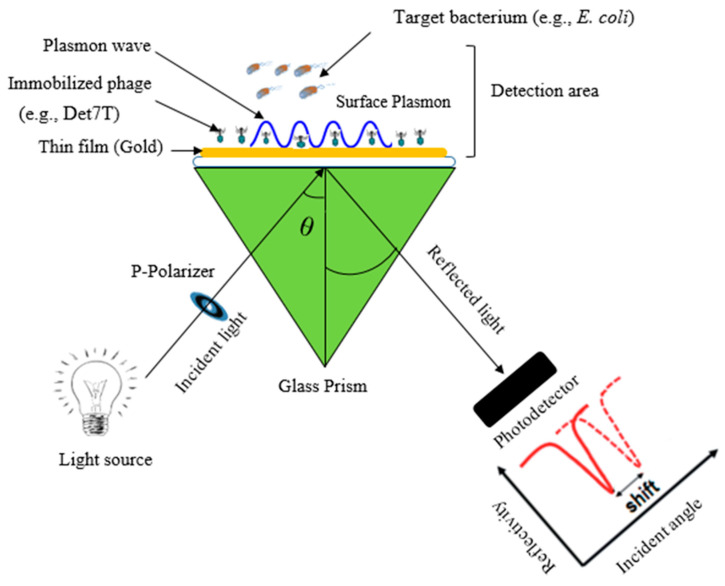
Schematic diagram illustrating the working principle of a surface plasmon resonance sensor (SPR) using bacteriophages.

Surface plasmon resonance (SPR) sensors are optical sensors that use distinct plasmon electromagnetic waves to detect (quantify) analytes based on molecular interactions with the biosensor. SPR biosensing, as a spectroscopic method, allows the real-time and quantitative detection of the binding agents or molecules freely without any kind of labeling [7]. The optical system of this type of biosensor consists of a light-emitting diode (LED), a photodiode array, a glass prism, and an optical surface. The molecular networking at the surface of this sensor drives angular changes in the reflected light, which changes the refractive index (Figure 4). The photodiode array detects the shift in angle and provides the result as a response unit (RU), which is equivalent to the whole mass of the bound ligands [75]. Foodborne microbes can be detected using binding proteins from bacteriophages and the phages themselves, which are incorporated into the SPR sensor system as biosensors. For instance, Singh and colleagues utilized the tail spike protein of an engineered phage (P22) immobilized onto a gold surface for the accurate and fast detection of *Salmonella* with a sensitivity of 10^3^ CFU/mL [52]. Choi et al. isolated a novel bacteriophage, KFS-SE2, from an eel farm for the detection of *Salmonella* Enteritidis on a food sample using the SPR platform. However, detailed information about its application in food has not been demonstrated [17].

Shin and Lim developed a novel 6HN-J-functionalized SPR biosensor comprising a segment of tail fiber protein derived from the lambda phage. This biosensor provided the fast, label-free detection of *E. coli* K-12 in the range of 2 × 10^4^–2 × 10^9^ CFU/mL and showed a lower detection limit of 2 × 10^4^ CFU/mL within 20 min [76]. However, the researchers reported a nonspecific binding with *P. aeruginosa.* The SPR sensor has also been shown to be efficient in the detection of methicillin-resistant *S. aureus* (MRSA), *E. coli* O157:H7 [77], *E. coli* K12, *S. aureus* [78], and hepatitis B virus (HBV) [79]. *S.* Typhimurium has been detected by an SPR device prepared via the immobilization of full-length engineered Det7 phage tail proteins (Det7T) on gold-coated surfaces by amine-coupling. This platform was able to detect *S.* Typhimurium quickly (within ~20 min) with a detection limit of 5 × 10^4−5^ CFU/mL in 10% apple juice and water [80].

#### 5.1.2. Bioluminescence Sensors

A bioluminescence sensor relies on the enzymatic (luciferase) cleavage of an organic compound, luciferin, which ultimately emits light in a living organism (especially in *Vibrio* strains). The ATP bioluminescence tests are a fast, sensitive and uncomplicated approach to the detection of bacterial contamination. In this assay, the cytoplasmic ATP released from a lysed bacterial cell is measured by the luciferase bioluminescence reaction [14].

Several studies have shown that different types of bioluminescence that have been obtained from different organisms can be integrated into the genome of bacteriophages for the quick and efficient detection of pathogens from food samples. For instance, the light-emitting features (luminescence values) of the NanoLuc luciferase (NLuc) reporter phage was designed by incorporating luciferase coding sequences derived from other organisms such as cnidarians, bacteria, and crustaceans into the genes of the *Listeria* phage A500 (A500::nluc ΔLCR), and the signal was found to be 100-fold higher than those of the other reporters. Hence, the NLuc luciferase-based assay is sensitive and able to directly detect as low as 3 CFU/100 mL *L. monocytogenes* in lettuce and milk samples, 72 h faster than culture-based approaches [14]. In a related study, a set of T7-based phages encoding an NLuc carbohydrate-binding module fusion protein (NLuc-CBM) were used for the detection of *E. coli* in water with a detection limit of 1 CFU/100 mL in less than 10 h [20,81].

In a study by Zhang et al. [56], a reporter phage was designed to detect *E. coli* O157:H7 in food samples. In this assay, the genome of the *E. coli* phage, ΦV10, was modified by incorporating a specific bioluminescent, Nluc, which is derived from *Oplophorus gracilirostris* (deep-sea shrimp), coupled with the commercial luciferin (Nano-Glo^®^). At a 1.76 × 10^2^ pfu/mL concentration of the reporter phage, the assay enabled the detection of 5 CFU of *E. coli* O157:H7 grown in Luria–Bertani broth within 7 h. A comparable detection was obtained using ΦV10 reporter phages in ground beef at 9.23 × 10^3^ pfu/mL within a 9 h turn-around time [56].

Kim and colleagues developed a bioluminescence sensor using an engineered reporter phage, SPC32H-CDABE, at a minimum detection limit of 20 CFU/mL of *Salmonella* within 2 h, and the signals raised at a parallel rate to the concentration of contaminated bacteria found in milk, lettuce, and sliced pork [82]. The researchers proclaimed the sensor to be a promising diagnostic tool for the detection of *Salmonella* contamination in food [82]. In another study, a substrate-independent luminescent phage-based biosensor was developed using the HK620 and HK97 bacteriophages for the detection of enteric bacteria such as *E. coli* in water samples. The developed bioluminescence was specific and allowed the detection of 10^4^ bacteria/mL in 1.5 h post-infection without the need for enrichment or a concentration step [83].

#### 5.1.3. Fluorescent Bioassay

Phage-based fluorescent bioassays have also been combined with fluorescently labeled bacteriophages that are involved in binding and detecting the host bacterium. An epifluorescent filter technique or flow cytometry has been used to detect phage–bacteria interactions. The reported sensitivity of this assay is about 10^2^–10^3^ CFU/mL and 10^4^ CFU/mL for epifluorescent and flow cytometry microscopy detection, respectively [84].

Vinay and co-workers demonstrated the detection of enteric bacteria such as *E. coli* and *S.* Typhimurium in water using phage-based fluorescent biosensor prototypes developed using the intact temperate phages HK620 and P22, respectively. The method is robust, fast, and sensitive, enabling the detection of as low as 10 bacteria/mL without enrichment or a concentration step [85]. Table 1 summarizes the use of different phage-based biosensor techniques for foodborne bacterial pathogens.

**Table 1:** Optical phage-based biosensors.

Transducer	Host Bacterium	Bio-Receptor (Phage)	LOD CFU/mL	Assay Time	Food Samples	Ref.
SPR sensor	Methicillin-resistant *Staphylococcus aureus* (MRSA)	BP14	10^3^	NR	NR	[77]
	*Salmonella* spp.	P22	10^3^	3 min	Chicken carcass (wash)	[52]
	*S. aureus*	12,600	10^4^	NR	NR	[86]
	*Campylobacter jejuni*	NCTC 12,673 TSP	10^2^	45 min	Milk	[87]
	*E. coli* O157:H7	T4	10^3^	NR	Skim milk	[88]
	*E. coli* K12	T4	7 × 10^2^	NR	Skim milk	[89]
	*L. monocytogenes*	scFv	2 × 10^6^	NR	NR	[90]
	*S. aureus*	12,600	10^4^	NR	NR	[86]
Bioluminescence sensor	*E. coli* G2-2	AT20	10^3^	NR	NR	[42]
	*E. coli*	*E. coli* phage	10^3^	60 min	NR	[91]
	*Salmonella* Newport	Newport (Felix) phage	10^3^	NR	NR	[91]
	*Salmonella* Enteritidis	SJ2	10^3^	120 min	NR	[42]
	*E. coli*	Wild-type and modified T4	6 × 10^3^	NR	NR	[39]
	*Yersinia pestis*	Phage A1122 with lux tag	10^2^	NR	NR	[92]
	*E. coli* B	lacZ T4 phage	10	NR	Water	[93]
	*P. aeruginosa*	Pap1	56	NR	Milk	[94]
	*S. flexneri*	Shfl25875	10^3^ CFU/g	NR	NR	[95]
Fluorescent bioassay	*E. coli*	T7	20	NR	NR	[30]
	*E. coli*	QD-labeled lambda phage	ND	NR	NR	[96]
	Staphylococcal enterotoxin B (SEB)	phage-displayed peptides	1.4 ng	NR	NR	[97]
	*E. coli* O157:H7	PP01	1	NR	Apple juice	[98]
	*S. aureus*	P-*S. aureus*-9	2.47 × 10^3^	NR	NR	[99]
	*S.* Typhimurium	P22	1 CFU/24 mL	NR	Milk	[100]
	*B. anthracis*	Wβ	10^4^ CFU/g	NR	NR	[101]
	*E. coli* TD2158	HK620	10^2^–10^4^	NR	NR	[85]
QCM-based assays	*Salmonella* Typhimurium	Filamentous phage	10^2^	3 min	NR	[102]
	*M. tuberculosis* and *M. smegmatis*	D29	10^3^	NR	NR	[103]
	*S. aureus*	12,600	10^4^	NR	NR	[104]
	*E. coli*	T4	NR	NR	Milk	[16]
	*E. coli* K12	Wild type	10^3^	NR	NR	[105]

NR—not reported.

### 5.2. Phage-Based Electrochemical Biosensors

Phages are specific to their host organisms and can act as transducers for electrochemical sensors. In a phage-based electrochemical biosensor, an electric current applied from an external source is used to attach the phage in an appropriate orientation. Richter et al. immobilized a T4 phage on a gold surface with the aid of 10 volt electric power for 30 min and observed a four-fold rise in the sensitivity of the ordered phage sensor compared with the disordered one [106]. They also suggested that the Debye length (L_D_) between the sample solution and the sensor’s surface is crucial for the successful alignment of bacteriophages. A 33-fold rise in the density of phages on the surface compared to the chemical modification of the surface with dithiobis succinimidyl propionate (DTSP) and the sensitivity of the sensor increased by 64-fold in comparison to the physical adsorption immobilization method [107]. A typical phage-based electrochemical sensor consists of potentiometric and amperometric measurements [108]. Table 2 summarizes the different foodborne bacteria that have been detected using different types of phage-based electrochemical biosensors.

#### 5.2.1. Amperometric Biosensors

Phage-based amperometric biosensors are one of the electrochemical sensors that have received much attention due to their simplicity, high sensitivity, specificity, and suitability for field testing. However, inhibitors can interfere with the assay and lower its specificity. In this platform, the phages are used either as a probe for the detection of a target bacterium or as a lysing agent for the indirect detection of pathogens using the metabolites released from the lysed cells [25]. Amperometric biosensors have been developed to quantify the flow of the current between electrodes when the oxidation–reduction reaction takes place. In this assay, enzymes such as horseradish peroxidase (HRP), glucose oxidase, and alkaline phosphatase (AP) are used as bio-receptors [109].

Several phage-based amperometric biosensors have been introduced for the detection of foodborne bacterial pathogens from food surfaces. Neufeld et al. designed phage-based amperometric techniques (specifically, β-D-galactosidase) for the detection of *E. coli* at concentrations as low as 1 CFU/100 mL within 6 to 8 h [33]. Likewise, Yemini et al. used the same platform to detect *M. smegmatis* and *B. cereus* using β- and α- glucosidases, respectively, as markers with a detection limit of 10 CFU/mL within 8 h [34]. Xu et al. designed a T4 phage-based sensor with a micro-gold electrode for the detection of *E. coli* from unspecified food samples. The sensitivity of this amperometric biosensor is in the range of 1.9 × 10^1^–1.9 × 10^8^ CFU/mL of the bacterial cells [25].

Quintela and Wu developed a portable sandwich-type phage-based amperometric biosensor using the environmental phage isolates belonging to the *Myoviridae* and *Siphoviridae* families. The sensor was highly specific to various Shiga toxin-producing *E. coli* (STEC) serogroups. The amperometric biosensor showed a detection limit of 10–10^2^ CFU/mL for the STEC O26, O157, and O179 strains within 1 h [15]. In another study, Nikkhoo et al. introduced a quick and inexpensive bacterial detection platform using T6 bacteriophages in combination with ion-selective field-effect transistors (ISFETs) and potassium-sensitive membranes (potassium ion detection). This amperometric platform was highly specific for the detection of *E. coli* in less than 10 min [110].

#### 5.2.2. Electrochemical Impedance Spectroscopy (EIS) Biosensors

**Figure 5:**
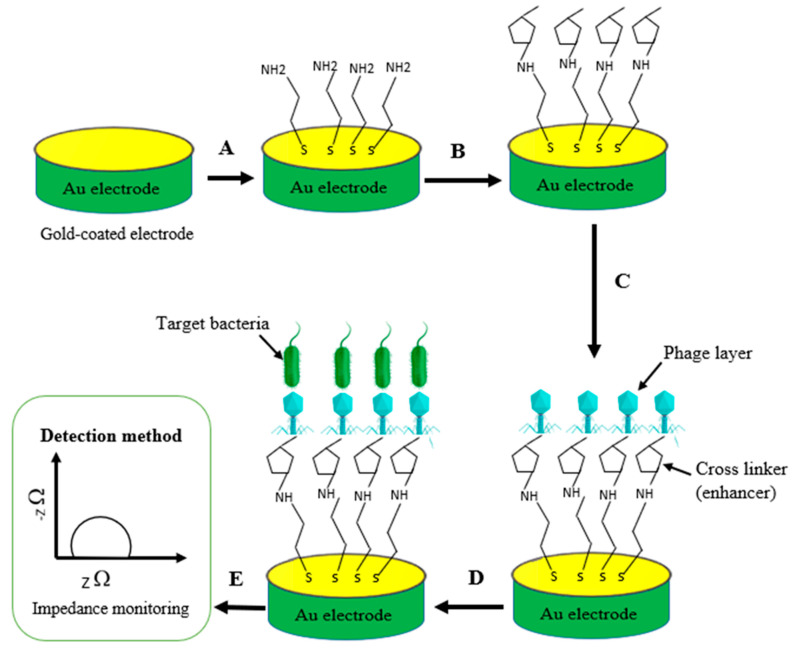
Schematic illustration of phage-based impedimetric biosensor, showing steps involved in phage immobilization and target bacteria detection: (**A**) surface modification of gold electrode using chemical linker (e.g., cysteamine); (**B**) cross-linker/enhancement using 1,4 dithiocyanate (PDICT); (**C**) immobilization of phages and treatment with ethanolamine to block nonspecific binding; (**D**) capture of target bacteria; and (**E**) impedance measurement (detection of target pathogen).

Electrochemical impedance spectroscopy (EIS) is a novel biosensor that uses functional sinusoids. The analysis is carried out based on changes in the electrical impedance (conductance, impedance, and capacitance) of the medium (Figure 5). The microbial metabolism in the medium reduces the capacity of the impedance [111]. Bacteriophages immobilized on an electrode are used as probes in this platform to detect bacterial strains at the electrode’s surface [112]. This technique is applicable for the detection of *E. coli* in inoculated samples or pure culture media ranging from 10^4^ to 10^7^ CFU/mL [113]. Webster et al. designed a phage-based impedimetric microelectrode array biosensor. The results indicated that the sensitivity of the impedimetric biosensor was enhanced by reducing the gap and width of the electrode and by using a lower relative dielectric permittivity [114]. An impedimetric biosensor (a label-free system) was proposed by Tlili et al. for the analysis of *E. coli* B with T4 phage-based EIS by covalently immobilizing them on a gold surface (cysteamine-modified) with a detection limit of 8 × 10^2^ CFU/mL in less than 15 min [115]. A screen-printed graphene sensor surface (electrode) was immobilized by highly specific lytic phages for the quick detection of *Staphylococcus arlettae* [116]. Table 2 summarizes some of the foodborne pathogens that have been detected using this technique.

**Table 2:** Some examples of phage-based electrochemical sensors.

Transducer	Phage	Host Bacterium	Food Samples	LOD (CFU/mL)	Ref.
Impedimetric Sensors	T4	*E. coli* K12	NR	10^4^	[117]
	T4	*E. coli* K12	NR	10^4^	[118]
	T4	*E. coli* K12	NR	10^3^	[105]
	Gamma phage	*B. anthracis*	Water	10^3^	[119]
	T4	*E. coli B*	Water	8.0 × 10^2^	[115]
	Specific phage	*S. arlettae*	NR	2	[116]
	T4	*E. coli* K12	NR	10^2^	[120]
	T2	*E. coli* B	NR	10^3^	[121]
	CBD	*Listeria*	NR	1.1 × 10^4^	[122]
	Endolysin Ply500	*L. monocytogenes*	Milk	10^5^	[122]
	Lytic phage	*Salmonella* Newport	NR	10^3^	[123]
Amperometric Biosensors	T4	*E. coli*	NR	1	[124]
	Phage lambda	*E. coli*	NR	1 CFU/100 mL	[33]
	M13	*E. coli* TG1	NR	1	[125]
	B1-7064	*B. cereus*	NR	10	[34]
	D29	*M. smegmatis*	NR	10	[34]
	T7	*E. coli*	NR	10^2^	[126]

NR—not reported.

### 5.3. Micromechanical Biosensors

#### Phage-Based Quartz Crystal Microbalance Assays

A phage-based quartz crystal microbalance (QCM) sensor is used to quantify the mass of analytes via immobilized phages on the surface of a sensor that is made from quartz crystal [127]. The quartz crystal fluctuates by an alternating current (AC current) at a specific resonance frequency. The frequency of the resonance is dependent on changes in the surface mass [128]. The phage-based QCM assays enhance the deposition of bacterial cells by capturing various components of the phage and ultimately changing the mass on the sensor surface. Guntupalli et al. used the phage 12,600 as a sensor (probe) in a phage-based QCM assay [104]. Olsen and co-workers developed a filamentous phage-based sensor that adsorbed ~3 × 10^10^ phages/cm^2^ physically on a piezoelectric transducer surface, which enabled the fast detection of *S*. Typhimurium. This phage-based QCM sensor exhibited a low LOD of 10^2^ CFU/mL with an assay time of <3 min [102].

### 5.4. Phage-Based Magnetoelastic Biosensor

**Figure 6:**
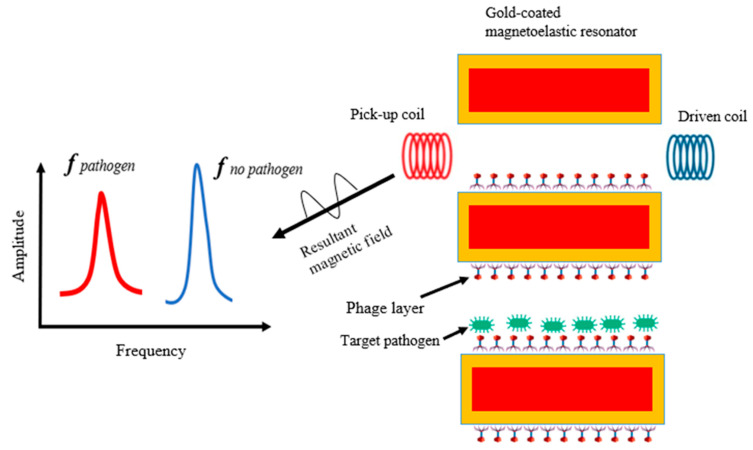
Schematic diagram elucidating the detection principle of the magnetoelastic phage sensor platform.

Phage-based magnetoelastic (ME) sensors use a wireless, mass-sensitive technique for the simple, specific, and rapid detection of biological analytes such as *B. anthracis* spores, *Salmonella*, and *E. coli* cells on food surfaces [129]. This biosensor consists of a magnetoelastic resonator immobilized with phages that act as bio-probes to recognize the target organism [23]. This sensor detects pathogens by measuring changes in the resonant frequency, which is proportional to changes in the sensor’s mass (Figure 6). An ME biosensor is a simple, time-effective, and cost-effective detection platform for foodborne pathogens in different food matrices, and can be a substitute for the qPCR method [130]. This biosensor has been used to detect *S.* Typhimurium directly on the shells of eggs and various fresh produce surfaces, including tomatoes, spinach leaves, and watermelons [131]. Wang et al. fabricated an ME using filamentous E2 phages specific for the detection of *S.* Typhimurium on fresh spinach leaves. The bacterium was detected after a minimum incubation time of 7 h with a detection limit of 100 CFU/25 g [131]. In another study, Chen et al. developed an ME biosensor for the detection of *Salmonella* using the phage C4-22 from the surface of chicken breast fillets in 2–10 min with a detection limit of 7.86 × 10^5^ CFU/mm^2^ [132]. A ferromagnetoelastic biosensor was designed using a tailed *B. cereus*-specific phage as a novel biorecognition tool for the detection of *B. cereus* in food matrices; however, the application of this biosensor in food samples has not been explored yet [18]. In general, ME biosensors show excellent specificity and sensitivity in pathogen detection and can be used for the real-time detection of target pathogens [132]. Table 3 summarizes the different foodborne bacteria that have been detected using the different types of phage-based micromechanical biosensors.

## 6. Conclusions and Future Directions

Bacteriophages have very important characteristics that make them ideal biorecognition agents for incorporation into biosensors for the detection of foodborne bacterial pathogens in food samples. They are highly specific; therefore, phage-based sensors are unaffected by background flora. As phages infect only living host bacteria, a phage-based sensor can easily distinguish living from dead organisms. The resistance of phages and phage-associated proteins to a wide range of temperatures, pH values, and organic solvents makes phage-based biosensors superior to other conventional pathogen-detection techniques. Generally, bacteriophage-based biosensor systems are cost-effective, specific, and more stable than conventional foodborne pathogen detection techniques. Unlike antibodies, bacteriophages can be produced in large quantities readily; thus, the fabrication of a biosensor using whole phages or phage proteins could be a cost-effective economical platform [137]. Currently, new phages with multiple binding sites on their surface or with other desirable properties can be generated using advanced synthetic biology approaches. This enables phages to be used for a wide range of biosensor applications [12].

With all the advantages mentioned above, there are certain challenges related to the development of phage-based biosensors that need attention. An obvious challenge of phage-based biosensors is the employment of bacteriophages that have a broad host range in a manner so that false-negative results can be avoided. Bacteriophages typically detect a specific receptor on the host cell’s surface; therefore, phage-based sensors must be tested against target and nontarget bacteria to diminish the chance of false-negative results. Besides, bacterial contamination or the presence of lipids, carbohydrates, and proteins could profoundly affect the binding efficiency and phase immobilization on the sensor surface. In addition, phage resistance is an emerging challenge due to the lack of receptors on the surface of the host organism required for phage adsorption, or host resistance triggered by eliciting intracellular defense mechanisms. This phenomenon can also affect the development of phage-based biosensors. However, such a problem can be overcome by using a “phage cocktail” containing a mixture of phages. The idea of a phage cocktail has to be adopted in future phage-based biosensor application platforms, especially for the simultaneous detection of multiple foodborne pathogens [14].

Another challenge for the establishment of a stable phage-based biosensor is the formation of stable chemical bonds between the surface of the biosensor and the phage attachment domain. For this, the physical as well as chemical features of the phages have to be explored in-depth to continue with suitable reactions to generate a stable sensor platform [138]. In addition, it has been recognized that when phage-based biosensors are exposed to a dry environment, the tail fibers lose their structural integrity, affecting bacterial capture on the sensor platform [52]. Nevertheless, engineered phage-based biosensors can circumvent these limitations. Engineering bacteriophages is inherently challenging due to the compact nature of the genomes and the availability of fewer noncoding sequences or restriction sites. However, with the development of numerous DNA synthesis methodologies and their application in synthetic biology, these drawbacks are likely to fall away rapidly. Hence, even with the development of synthetic biology, there is still a need for more insight into the genetic makeup of phage genomes that can be used for this purpose [138].

The selection of bacteriophages of a desired size, especially for nano-biosensor platforms, and the optimization of the expression of the binding domains on the surface of the phages remain the major challenges. In addition, the ability of phages or their proteins to be immobilized on the surface of sensor platforms through chemical anchoring or physical absorption is well developed; however, their stable attachment on other surfaces is a fertile topic for research exploration [12].

While the specificity of bacteriophages towards a host/target bacterium is the basis for the development of phage-based biosensors, there is a need for broadening the detection range for multi-pathogen detection. Introducing polyvalency to RBPs has become relevant to establishing a multiplexed platform for the rapid detection of foodborne pathogens, which is an area that has yet to be addressed [139].

In this review, a description of nearly 54 biosensors has been summarized. Though the detection limits and validation with samples for the majority of these sensors are known (Table 1, Table 2 and Table 3), many researchers failed to provide such information. These might be due to the lysis of the target bacterium by bacteriophages or interference from the food samples, which consequently could have obscured the accuracy of bacterial counts. In addition, the drying of biorecognition molecules on the sensor platform may have resulted in the loss of the captured target bacterium, which could have also affected detection. To overcome such limitations, genetically modified phages and/or advanced functional surface chemistry can be employed for stable phage immobilization.

Phage-based biosensors have been demonstrated to have great potential in the detection of pathogenic bacteria from food and the environment. However, the transition from the laboratory bench to commercial spaces has been very slow due to several constraints, including, but not limited to, a weak signal-to-noise ratio, sensitivity, and specificity of the bacteriophages; reproducibility; a short shelf-life of the sensor; instrument design; and cost.

The future advancement of phage-based biosensing platforms should also consider the development of new recognition platforms, improvements to signal amplification, and the establishment of nanostructures for the precise geometry of the sensor design. To this end, genetically modified phages are relevant since they can produce the desired peptides and proteins on their surface to generate an appropriate and multifunctional biorecognition platform. Furthermore, one of the most promising future directions of phage-based sensing is its compatibility with emerging biomolecules and nanostructures (quantum dots; metallic, magnetic, and polymer nanoparticles; etc.) to generate new and innovative phage-based nanodevices or bioinspired sensor tools. Such hybrid versatile sensors are well-suited for the detection of a wide variety of foodborne pathogens from various sources.

In conclusion, even though the progress made so far has been inspiring, the future of phage-based sensing still requires a strong collaborative effort between researchers working in diverse disciplines, such as molecular biology, microbiology, biochemistry, engineering, material science, biology, physics, and chemistry, to enhance the overall detection efficiency of the sensors. Moreover, care must be taken to avoid any potential public health hazards associated with the bacteriophages and the spread of the parental host (pathogenic bacteria) during bacteriophage production, purification, and storage.

## Figures and Tables

**Table 3:** Phage-based quartz crystal microbalance (QCM) and magnetoelastic (ME) biosensors.

Transducer	Host Bacterium	Phage	Food Sample	LOD (CFU/mL)	Assay Time	Ref.
Magnetoelastic	*S.* Typhimurium	E2	NR	5 × 10^2^	NR	[133]
	*S.* Typhimurium	E2	Romaine lettuce	5 × 10^2^	NR	[134]
	*B. anthracis*	JRB7	NR	NR	NR	[135]
	*S.* Typhimurium	NR	NR	1.5 × 10^3^ CFU/mm^2^	NR	[136]
QCM-based assays	*S.* Typhimurium	Filamentous phage	Chicken wash	10^2^	3 min	[102]
	*M. tuberculosis* and *M. smegmatis*	D29	NR	10^3^	NR	[103]
	*S. aureus*	12,600	NR	10^4^	NR	[104]
	*E. coli*	T4	Milk	NR	NR	[16]

NR—not reported.

## Data Availability

Not applicable.
